# Subocclusive ileus following off-label high-dose semaglutide use

**DOI:** 10.1530/EDM-25-0081

**Published:** 2025-11-26

**Authors:** Arina Nikolaeva, Martin Vandeputte

**Affiliations:** ^1^ Université Libre de Bruxelles, Faculty of Medicine, Brussels, Belgium.; ^2^ Hôpitaux Iris Sud HIS , Internal Medicine, Brussels, Belgium.

**Keywords:** ileus, high-dose, semaglutide, off-label

## Abstract

**Summary:**

Semaglutide, a GLP-1 receptor agonist (RA), is approved for the treatment of type 2 diabetes and obesity. However, its increasing off-label use, particularly for cosmetic weight loss, raises concerns considering its potential adverse effects. We report the case of a 37-year-old woman with normal body mass index who developed subocclusive ileus following unsupervised use of semaglutide.

**Learning points:**

- Our case highlights that semaglutide can lead to functional intestinal obstruction.
- Clinicians should maintain a high index of suspicion for GLP-1 RA related gastrointestinal complications.

## Background

GLP-1 receptor agonists, such as semaglutide, have emerged on the market as effective treatments for type 2 diabetes and, more recently, for weight management in people with obesity. These agents exert their effect by enhancing insulin secretion, suppressing glucagon, and delaying gastric emptying, thereby promoting satiety and glycemic control. Although generally well tolerated, gastrointestinal side effects such as nausea and constipation are common. Recently, concerns have emerged about their potential association with more serious gastrointestinal complications, including intestinal obstruction. While several large-scale studies and pharmacovigilance reports have evaluated this risk with various GLP-1 RAs, direct associations with semaglutide remain limited. This case adds to the growing body of evidence by presenting a gastrointestinal motility complication occurring after high-dose and unsupervised use of semaglutide.

## Case presentation

A 37-year-old woman was admitted to the Internal Medicine Department with a 5-day history of diffuse abdominal pain, diarrhea, and vomiting occurring more than four times per day. Systemic review also reported mild headaches over the preceding 2 days. She reported self-injecting 4.8 mg of subcutaneous semaglutide on two occasions over 2 weeks to lose weight, motivated by body image concerns, despite having a normal body mass index of 25 kg/m^2^. The medication was received from a friend and used without medical supervision or prescription. She had no known past medical history and was not taking any other medications. On examination, she was afebrile with normal cardiopulmonary findings. Abdominal exam revealed a soft but tender periumbilical region, diminished bowel sounds, and no peritoneal signs. No peripheral edema was observed.

## Investigation

Laboratory workup showed leukocytosis (WBC: 11.89 × 10^3^/μL), mildly decreased estimated glomerular filtration rate (eGFR: 57 mL/min/1.73 m^2^), and elevated lactate dehydrogenase (LDH: 322 U/L), while CRP, electrolytes, and liver enzymes remained within normal limits.

**Figure 1:**
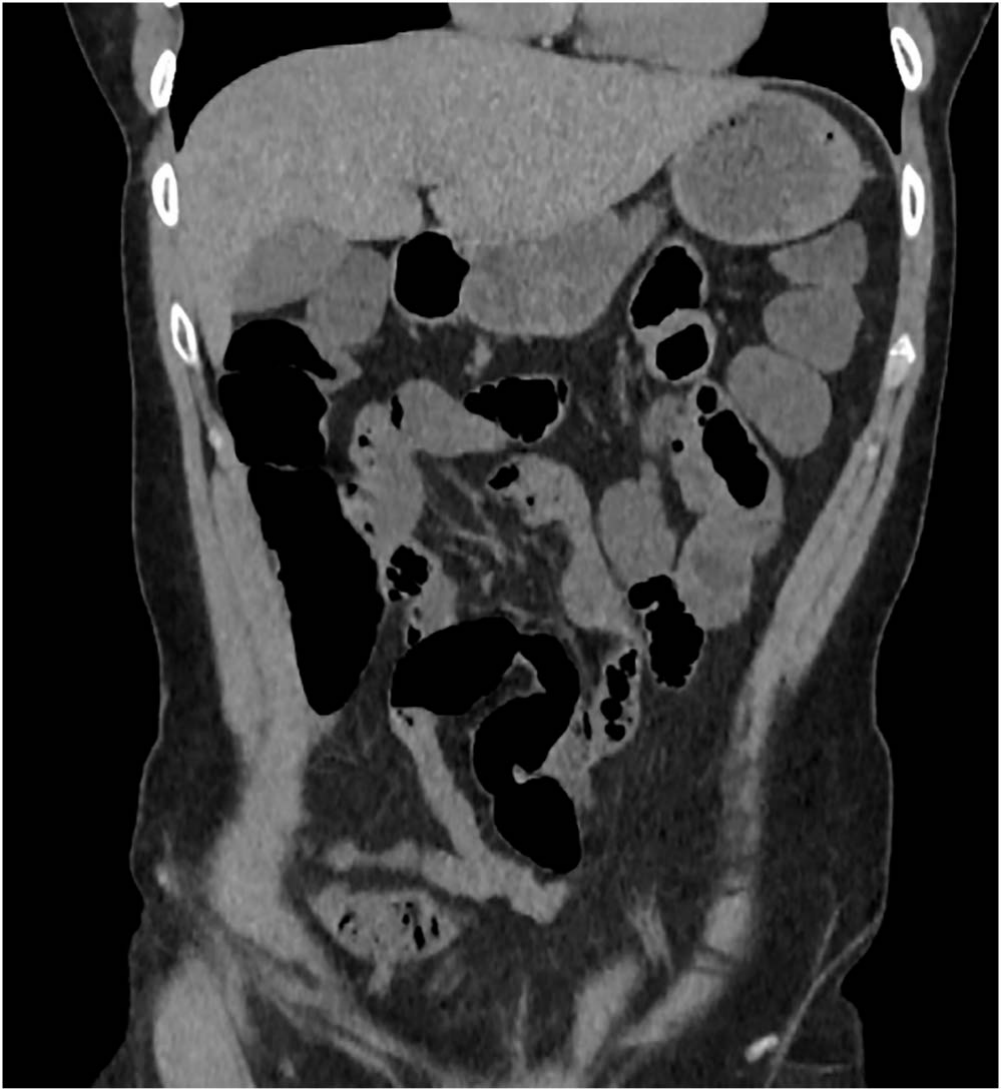
Abdominal CT showing dilated bowel loops with a transition point and no mechanical cause of obstruction.

Her symptoms persisted despite analgesics, prompting abdominal CT imaging. This revealed a subocclusive ileus characterized by dilated small bowel loops with a sharp transition to collapsed segments in the suprapelvic region, without an evident mechanical obstruction (Fig. 1).

## Treatment

The patient was managed conservatively with fasting and nasogastric tube decompression for 24 h. She received intravenous fluids, paracetamol 1 g IV twice daily, and metoclopramide for 2 days. A mental health and eating disorder assessment was proposed in order to evaluate potential risk factors related to body image and misuse of incretin-based therapy. However, the patient declined any psychological evaluation, as she did not perceive any need.

## Outcome and follow-up

Oral intake was resumed 24 h post-admission with gradual symptom resolution. Renal function normalized within 48 h. The nasogastric tube was removed after 1 day, and hospitalization lasted for 3 days in total. The patient has not resumed semaglutide use since discharge, and no relapse was observed at the 3-week follow-up consultation.

## Discussion

This case presents a rare but potentially serious gastrointestinal complication associated with semaglutide use in a patient without clinical indications at supratherapeutic doses. GLP-1 RAs are known to delay gastric emptying and slow intestinal transit (1). While these pharmacodynamic effects are beneficial in managing diabetes and obesity, they may predispose certain individuals to gastrointestinal motility disorders, particularly when used at high or inappropriate doses.

Although bowel obstruction has been reported with incretin therapies, most evidence involves liraglutide and, more recently, tirzepatide. In contrast, semaglutide has been less frequently implicated. This difference may partly reflect its shorter duration of clinical use and the fact that earlier studies typically involved doses up to 1 mg weekly for diabetes, rather than the higher 2.4 mg weekly regimen now approved and widely prescribed for obesity (2). Our case is particularly notable as the patient self-administered a supratherapeutic dose of 4.8 mg without the recommended dose titration. This excessive exposure likely contributed to the development of subocclusive ileus. Despite the growing use of GLP-1 RAs, long-term safety data, particularly regarding gastrointestinal motility, remain limited (3).

The risk of bowel obstruction caused by GLP-1 RAs is controversial. A large Nordic study using nationwide data from Sweden, Denmark, and Norway (2013–2021) compared over 120,000 GLP-1 RA users to 185,000 SGLT-2 inhibitor users, and no significant difference in bowel obstruction risk was found. Interestingly, a nonsignificant trend suggested a protective effect with GLP-1 RAs (4).

In contrast, recent large-scale studies reported opposing findings. A UK-based cohort study comparing over 25,000 patients treated with GLP-1 RAs to 67,000 patients on SGLT-2 inhibitors found that GLP-1 RA use was significantly associated with a higher risk of intestinal obstruction, observed around 1.6 years of treatment. Particularly, semaglutide was the only GLP-1 RA with no recorded obstruction events (5).

A pharmacovigilance review of over 500,000 adverse drug reactions found 698 cases of intestinal obstruction, with 64.8% linked to incretin-based therapies. While GLP-1 RAs accounted for nearly half of these cases and showed more than fourfold reporting odds ratio, semaglutide was only implicated once (6).

Finally, a PharMetrics Plus database study analyzed more than 5,000 patients with obesity treated for weight loss, mainly with liraglutide (76.6%), semaglutide (11.3%), and bupropion-naltrexone (12.1%). GLP-1 RA use was linked to a significantly increased risk of bowel obstruction. Notably, bowel obstruction events were reported in 8% of patients treated with liraglutide, whereas no such events were observed among those receiving semaglutide (7).

The potential mechanism of action of this effect is that GLP-1 RAs delay gastric emptying and slow small bowel transit by acting on the enteric nervous system, particularly via postganglionic neurons and muscarinic receptors, disturbing coordinated bowel motility (8, 9). These effects, although therapeutic in glycemic control, may lead to significant motility disturbances in susceptible individuals, as reflected in recent clinical observations and pharmacovigilance reports. The limited number of reported obstruction cases with semaglutide may reflect its more recent introduction, the lower doses used in early clinical trials, and the smaller proportion of exposed patients compared to other incretins.

To conclude, this case demonstrates that semaglutide itself may induce subocclusive ileus, despite prior studies suggesting a lower risk compared to other GLP-1 RAs. As off-label and unsupervised use of semaglutide increases, clinicians should be aware of this potential complication, even in healthy individuals without obesity or diabetes. Prevention through education and responsible prescribing remains relevant.

## Declaration of interest

There is no conflict of interest that could be perceived as prejudicing the impartiality of the research reported.

## Funding

This research did not receive any specific grant from any funding agency in the public, commercial, or not-for-profit sector.

## Patient consent

Written informed consent for publication of their clinical details was included and obtained from the patient.

## Author contribution statement

Both authors have contributed equally to the writing of this case report.

## Patient’s perspective

The patient initially felt anxious when her abdominal pain began and powerless when the analgesics failed to improve her condition. Upon learning her diagnosis and the role of semaglutide, she felt guilty for taking it without medical supervision. She was relieved when her abdominal pain resolved and went on to resume a normal diet. Following medical advice, GLP-1 RA was discontinued to avoid further side effects, which she accepted without any concerns.
